# Bispecific Antibodies and Antibody–Drug Conjugates for Cancer Therapy: Technological Considerations

**DOI:** 10.3390/biom10030360

**Published:** 2020-02-26

**Authors:** Hyunbo Shim

**Affiliations:** Department of Life Science, Ewha Womans University, Seoul 03760, Korea; hshim@ewha.ac.kr

**Keywords:** bispecific antibody, antibody–drug conjugate, cancer therapy

## Abstract

The ability of monoclonal antibodies to specifically bind a target antigen and neutralize or stimulate its activity is the basis for the rapid growth and development of the therapeutic antibody field. In recent years, traditional immunoglobulin antibodies have been further engineered for better efficacy and safety, and technological developments in the field enabled the design and production of engineered antibodies capable of mediating therapeutic functions hitherto unattainable by conventional antibody formats. Representative of this newer generation of therapeutic antibody formats are bispecific antibodies and antibody–drug conjugates, each with several approved drugs and dozens more in the clinical development phase. In this review, the technological principles and challenges of bispecific antibodies and antibody–drug conjugates are discussed, with emphasis on clinically validated formats but also including recent developments in the fields, many of which are expected to significantly augment the current therapeutic arsenal against cancer and other diseases with unmet medical needs.

## 1. Introduction

Therapeutic antibodies exert their effects by binding to and neutralizing extracellular target molecules. The advantages of therapeutic antibodies include strong and specific binding to the target antigen, thus maximizing efficacy and safety, and the ability to block protein–protein interaction, which, in general, is not attainable with small-molecule drugs. However, like most other therapeutic modalities, blocking a single disease-causing molecule by an antibody rarely cures complex diseases such as cancers. Few diseases are totally dependent upon one target and its signaling pathways, and the downregulation of the target molecule and/or the development of compensatory pathways by diseased cells often leads to drug resistance. For example, it has been suggested that the activation of ErbB3 or c-Met signaling contributes to the resistance of cancer cells to EGFR-targeting cetuximab [1,2].

In order to overcome the limitations of therapeutic antibodies and enhance their efficacy, various engineering and modification approaches have been devised and applied to the conventional immunoglobulin molecular format. Arguably the most prominent of these approaches are the bispecific antibody (bsAb) and antibody–drug conjugate (ADC) formats. The basic ideas behind these formats are quite straightforward: For bsAb, simultaneous engagement of two different targets by a single antibody-like molecule may have synergistic or emergent therapeutic effects, and for ADC, the cancer-selective delivery of potent cytotoxic payloads may eradicate target-expressing cancer cells while sparing normal healthy tissues. However, the implementation and clinical application of these novel formats require a considerable amount of molecular engineering efforts. Antibody physicochemical properties such as solubility, propensity for oligomerization/aggregation, and thermal and chemical stability, as well as their pharmacological characteristics in vivo, are all affected by combining two antibodies or their fragments, or by attaching cytotoxic payloads through chemical linkers.

In this review, the current state of the art of bsAb and ADC in cancer treatment is discussed. Various different formats of bispecific antibodies have been developed, including those with IgG-like configurations such as heterodimeric IgG, IgG-scFv, and DVD-Ig, as well as fragment-based designs like tandem scFv and diabody, all of which have different sets of development and production challenges. For ADC, issues such as conjugation site specificity, linker chemistry, cleavage strategy, and the potency of the payload need to be optimized to maximize therapeutic efficacy while minimizing adverse events. Despite all the challenges, a number of bsAbs and ADCs have been approved for cancer treatment and many promising candidates are currently in the late stages of clinical trials, as summarized in Table 1.

## 2. Bispecific Antibodies

**Figure 1:**
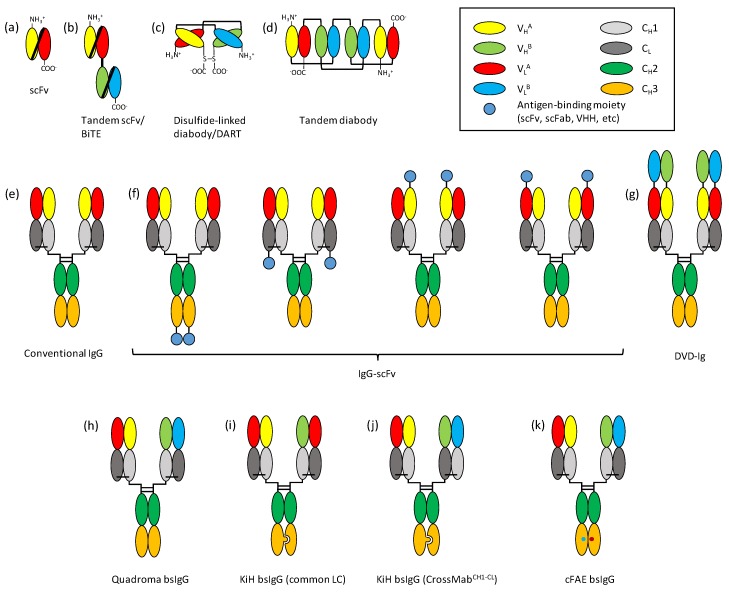
Schematic representations of bispecific antibody (bsAb) formats discussed in this article. (**a**) Single chain Fv (scFv), (**b**) tandem scFv format of bispecific T cell engager (BiTE), (**c**) disulfide-linked diabody format of dual affinity retargeting (DART) bsAb, (**d**) tandam diabody (TandAb), (**e**) conventional immunoglobulin G (IgG), (**f**) IgGs with additional binding units such as scFv, (**g**) dual variable domain immunoglobulin (DVD-Ig), (**h**) quadromab bsAb, (**i**) knobs-into-holes (KiH) bsAb with a common light chain, (**j**) KiH-CrossMab^CH1-CL^, and (**k**) bsAb by controlled Fab arm exchange (cFAE).

More than 100 different bsAb formats have been reported according to a recent review [3]. While their molecular architectures are different, virtually all of them can be grouped into one of the two large categories: fragment-based bsAbs and Fc-based bsAbs. Fragment-based bsAbs consist of two or more antibody fragments (usually scFv (Figure 1a), but also Fab or single-domain Ab (sdAb)) held together by a peptide linker, disulfide bonds, and/or noncovalent inter-domain interactions. Fc-based bsAbs have homo- or heterodimeric Fc domains (and less frequently CH3 domains only) to which Fab, scFv or sdAb are attached through a peptide linker. Additional antigen-binding moieties may also be present at either N- or C-terminus of any of the polypeptide chains comprising the bsAb, greatly increasing the structural diversity of bsAb design. Some of the bsAb formats discussed in this review are shown in Figure 1.

### 2.1. BsAb Formats

#### 2.1.1. Fragment-Based bsAbs

Fragment-based bsAbs, consisting exclusively of antibody variable domains, are a major class of bsAb formats. They are in general smaller in size than Fc-based bsAbs (see below) and hence, shows faster clearance and better tissue penetration in vivo, which significantly influence their pharmacological properties. Although these molecules are vaguely similar to one another in their structures with two or more antibody fragments connected by flexible peptide linkers, each of them has a unique molecular configuration, which results in differences in aspects such as physicochemical properties, biological activity, and production methods. While there are a variety of different fragment-based bsAb formats, three of them have been more extensively investigated in preclinical and clinical studies: tandem scFvs, dual affinity retargeting (DART) proteins, and tandem diabodies (TandAbs).

Tandem scFvs are one of the most well-studied bsAb formats, partly because of the first approved fragment-based bsAb blinatumomab (Blincyto™). Blinatumomab is a bispecific T-cell engager (BiTE) with VL_CD19_-(GGGGS)_3_-VH_CD19_-(GGGGS)-VH_CD3_-(GGGGS)_3_-VL_CD3_ configuration (Figure 1b) [4] and binds simultaneously to CD3ε on T-cells and CD19 on lymphocytes of B-cell lineage. By ligating T-cells with target cells and activating T-cells through CD3, blinatumomab can induce the killing of CD19-expressing B-cells including malignant B-cell leukemia and lymphoma [4]. The order of variable domains is critical for the production of functional BiTE [5], while the length of the inter-scFv linker may either be short (GGGGS) or long ((GGGGS)_3_) without significantly affecting its tumor-killing activity [6]. Of course, it is unlikely that there exists a single optimal molecular configuration for all tandem scFvs, because the effect of variable domain order on the functionality of scFv differs among antibodies, and close proximity of two scFvs connected by a short linker may interfere with the antigen binding activity of certain antibodies. Unlike more traditional antibody fragment formats, such as Fabs or scFvs that are produced from *E. coli*, BiTEs are usually produced from mammalian cells [4,6], presumably because of their rather complex tertiary structure and multiple disulfide bonds. Their small size (~60 kDa) and the lack of Fc domain make them rapidly cleared in vivo (*t*_1/2_ = 1.25 ± 0.63 h [7]) and necessitate administration by continuous infusion [8], which may be overcome by, e.g., fusion to the Fc domain [9,10,11]. On the other hand, the compact size of BiTEs seems to keep the distance between target and effector cells optimal for tumor cell killing by T-cells, at least in the case of blinatumomab [12]. Stability is another factor to consider in the development of BiTEs. While some BiTEs can be kept refrigerated for months in PBS without significantly losing binding activity [13], in clinical application, blinatumomab is stable for only up to 48 h at room temperature [14], possibly due to its scFv-based architecture. Despite the technical issues, BiTEs have a superb therapeutic efficacy, as proven by the success of blinatumomab, and more than 10 BiTE molecules are currently in the early stages of clinical evaluation for various hematological and solid cancers [15].

Dual Affinity Re-Targeting (DART) is another fragment-based bsAb design, distinct from the tandem scFv format of BiTEs. DART is a disulfide-linked diabody; i.e., two polypeptide chains in VH-linker-VL configuration form a heterodimer, with two cysteines at the opposing C-termini linked to each other by a disulfide bond (Figure 1c). Typically, to generate a DART, two polypeptide chains in, e.g., VL_A_-linker-VH_B_-tail and VL_B_-linker-VH_A_-tail configurations are co-expressed in a host cell. The use of a short linker (e.g., GGGGS) prevents the formation of monomeric scFv, and the tail sequence contains a cysteine residue for disulfide linkage. In theory, the co-expression of two such polypeptides could result in the formation of both heterodimer and nonfunctional homodimer. The introduction of heterodimerization sequences to the C-terminus of each chain can facilitate the formation of correctly paired DARTs, and affinity chromatographic purification using antigen-immobilized resin followed by size exclusion chromatography yields active heterodimeric proteins with high purity [16,17]. Unlike ordinary diabodies, which are held together by relatively weak non-covalent inter-variable domain interactions, the two polypeptide chains of DART are covalently linked. As a result, DARTs are highly stable, fully retaining their binding activity for up to 31 months at 4 °C in PBS and 8 weeks at 37 °C in human serum without a significant increase in aggregation [17]. Because Fv units of DART are maintained by noncovalent interdomain interactions between VH and VL without a peptide linker, they more closely resemble those of IgGs than scFv units of BiTE do. Moreover, the sequence and flexibility of the linkers as well as the relative orientation of two monospecific units differ between DART and BiTE [16]. Phase 1 clinical trial of duvortuxizumab (MGD011, a CD19×CD3 DART with Fc domain for longer serum half-life) has been terminated reportedly due to neurotoxicity and the competition from other CD19-targeting therapies [18]; however, there are a number of DART molecules currently undergoing clinical evaluation for indications such as colorectal cancer [19] and acute myeloid leukemia [20].

Tandem diabodies (TandAbs) are head-to-tail homodimers of two identical polypeptide chains in, e.g., VH_A_-VL_B_-VH_B_-VL_A_ configuration (Figure 1d). Unlike BiTEs and DARTs that are monovalent to each of their targets, TandAbs are bivalent to both antigens and thus, expected to have higher apparent affinity through the avidity effect. Kipriyanov et al. reported the construction and characterization of CD19×CD3 TandAbs in VH_CD3_-(9 a.a. linker)-VL_CD19_-(12 or 27 a.a. linker)-VH_CD19_-(9 a.a. linker)-VL_CD3_ configuration produced in *E. coli* [21]. The short 9–12 a.a. linker on both sides was employed to prevent the unwanted intrachain interactions of variable domains. For the middle linker position, the long 27 a.a. linker was designed to allow the structural flexibility required for the folding of TandAb and the antigen binding by the middle (second and third) Fvs, whereas the short 12 a.a. linker was expected to minimize the intrachain paring of variable domains while providing enough flexibility for folding and antigen binding. The construct with the 12 a.a. middle linker was solubly expressed in dimeric (i.e., TandAb) form; however, the 27 a.a. middle linker construct was produced predominantly as monomeric single-chain diabody in normal 2×YT medium due to the flexibility of the long linker. In another study, a CD19×CD3 TandAb with a short GGSGGS linker in all three positions was produced from mammalian cells [22]. The TandAb, AFM11, was reasonably stable and ~90% of the molecules remained unaggregated after seven days at 37 °C. At ~105 kDa, the molecular weight of TandAb homodimer is significantly higher than that of albumin (67 kDa) and the renal clearance rate of TandAb is expected to be much slower than those of smaller fragment-based bsAbs such as BiTEs or DARTs (~55 kDa). Indeed, AFM11’s serum half-life in phase I clinical trial was reported to be ~8 h [23], compared with ~2 h for blinatumomab [8]. For a fragment-based bsAb format TandAbs are stable and highly potent (see below in Section 2.2.2), and while AFM11 has been put on clinical hold after a fatal neurological adverse event was reported in phase 1 clinical trial, other TandAbs, including AFM13 (NK-cell engaging CD30×CD16A, phase 2, NCT02321592) and AFM24 (EGFRxCD16A, phase 1, NCT04259450) are being evaluated in clinical studies.

#### 2.1.2. Symmetric Fc-Based bsAbs

The fragment crystallizable (Fc) region is responsible for the antibody effector functions by binding to FcγRs and C1q, and also for the prolonged half-life of immunoglobulins through pH-dependent binding to FcRn [24]. Therefore, it is generally desirable for therapeutic antibodies to have an Fc region unless large size and longer half-life need to be avoided, and various engineering approaches have been applied to the Fc region for improved biological and physicochemical properties [25], including engineering for bispecificity [26]. Fc-based bsAbs can be categorized into two large groups: symmetric and asymmetric. Symmetric Fc-based bsAbs typically have additional Fv or scFv moieties at the N- and/or C-termini of the polypeptide chains, making them larger than conventional IgG antibodies (Figure 1e). On the other hand, asymmetric Fc-based bsAbs are produced by the preferential heterodimerization of two engineered Fcs, making them identical in size and shape to conventional IgG and each of the two arms of the bsAb recognizing a different antigen.

In symmetric Fc-based bsAbs, additional Fvs with second antigen specificity can be fused to either N- or C-termini of heavy or light chains of IgG, typically in the form of scFv (Figure 1f) [27] but also in linkerless Fv forms as in dual variable domain-IgG (DVD-IgG) (Figure 1g) [28]. Other antigen binding moieties, such as domain antibodies or alternative binding scaffold molecules, can also be utilized in place of scFv [29,30,31,32]. Attaching additional binding moieties to conventional IgGs is conceptually simple and straightforward, however, it may alter the physicochemical properties of the molecule significantly, depending on the properties of the added Fvs and the site of attachment. Therefore, such aspects of bsAb design as the fusion site (N- or C-termini, heavy or light chains), linker length and sequence, and the choice of the Fv as either main (IgG Fv) or appended (scFv) may need to be optimized for the practical implementation of this type of bsAbs [3,32,33,34].

The first study of IgG-scFv, using anti-dextran IgG with anti-dansyl scFv fused to the C-termini of CH3s through a GGGS linker [27], reported that the molecule retained the binding activity to FcγR and C1q as well as showing a longer serum half-life than F(ab’)_2_-scFv, although these Fc-mediated functions were significantly weaker than the IgG antibody without attached scFv. The anti-dextran and anti-dansyl affinities both decreased ~10-fold by the scFv fusion, and notably, the IgG-scFv could not mediate complement-dependent cytotoxicity toward dansyl-BSA-coated sheep RBC, possibly due to the altered orientation and flexible linker of the attached scFv that may interfere with C1q binding and/or the recruitment of the complement system. The apparent limitations of this initial IgG-scFv construct suggested that elaborate engineering of bsAb design was necessary in order to optimize their binding and effector functions.

In an effort to optimize the efficacy and physicochemical properties of IgG-scFv bsAbs, biparatopic anti-CCR5 IgG-scFv molecules with different sites of scFv attachment and/or varying lengths of the scFv linker were constructed, with and without an interdomain disulfide bond in the scFv moieties [34]. With conventional scFvs with a (G_4_S)_3_ linker and without an interdomain disulfide bond, a significant amount of aggregates (up to 50%) was observed at high concentration (≥1.0 mg/mL) and/or upon prolonged storage, which is a well-known, recurring problem for many scFv fusion proteins [35]. Introduction of a disulfide bond between V_H_44 and V_L_100 or a longer linker [(G_4_S)_4–6_] greatly improved the stability and monomer content of IgG-scFv, with some constructs showing properties comparable to conventional IgG molecules. In another study, Miller et al. observed that the disulfide-bond stabilization strategy may not be universally applicable, and the introduction of the interdomain disulfide bond resulted in lower expression and degradation of some scFvs [35]. In order to develop a more general approach to scFv stabilization, they constructed small (10~100 mutants) focused libraries of an anti-LTβR scFv, and screened them for target binding following thermal challenge. Stabilizing mutations were identified and combined to generate stabilized scFvs; IgG-scFv generated using the stability-engineered scFv showed a remarkable improvement in monomer content (~40% soluble aggregates for wild-type bsAb vs. ~7% for the stability-engineered IgG-scFv), demonstrating that the stability of the scFv moiety is a critical factor for the physicochemical properties of IgG-scFv bsAbs.

Instead of attaching scFvs, VH and VL can each be introduced to separate termini of different chains of IgG to form an additional Fv. A prominent example is DVD-Ig, in which a VH and a VL are attached to the N-termini of the heavy and light chains, respectively, resulting in a bsAb with two consecutive Fvs in each arm [28]. In this format, the presence of the distal Fv at N-terminus may interfere with the antigen binding by the proximal Fv [36], which can be partially relieved by linker engineering (see below). While glycine-rich flexible linkers are most commonly used to construct other types bsAbs, linker sequences that correspond to the N-terminus of CH1 or CL (and variations thereof) are frequently employed for the construction of DVD-Ig [28,37], presumably to facilitate the VH/VL heterodimerization by mimicking the variable/constant domain interfaces of natural antibodies. The crystal structure of IL12×IL18 DVD-Ig with such linkers (ASTKGP for V_H1_-V_H2_ and TVVAP for V_L1_-V_L2_) revealed that the distal Fv is positioned approximately 85° relative to the proximal Fv when the latter is occupied by the antigen (IL18), allowing efficient binding of both antigens by DVD-Ig [37]. In other DVD-Ig constructs, however, the presence of distal Fv may interfere with and compromise the antigen binding by proximal Fv; while this problem may be overcome by linker optimization or other engineering, an interesting approach took advantage of it by introducing an endopeptidase-susceptible sequence to one of the two linkers of DVD-Ig [36]. Selective cleavage of one linker provides increased flexibility of the distal Fv and stronger binding by the proximal Fv; it is plausible that such DVD-Ig can be engineered to be selectively activated by a cancer-specific endopeptidase in a prodrug-like manner.

Symmetric Fc-based bsAbs have relatively straightforward design principles, and tetravalency (bivalent to both targets) provide them with stronger binding to targets by the avidity effect; however, the presence of the appended binding units poses engineering challenges to develop therapeutic-quality molecules. A number of symmetric Fc-based bsAbs for cancer therapy have been tested or are currently in clinical trials: LY3164530 (cMET×EGFR IgG-scFv, phase I, NCT012221882) has completed phase I trial but further development was stopped due to significant toxicities and lack of a potential predictive biomarker [38], and ABT-165 (DLL4×VEGF DVD-Ig, phase 2, NCT03368859) [39] is being developed for metastatic colorectal cancer.

#### 2.1.3. Asymmetric Fc-Based bsAbs

IgG molecules are heterotetramers with C2 symmetry, consisting of two identical heavy and light chains each. The two identical heavy chains are held together by disulfide bonds at the hinge region and noncovalent interactions between CH3 domains, and each heavy chain is paired with a light chain by a disulfide bond between C-termini of CH1 and CL, as well as noncovalent interdomain interactions. Because the two Fab arms of conventional IgG are identical to each other, IgG is bivalent but monospecific. Asymmetric IgG-like bsAbs have essentially the same molecular architecture as conventional IgGs, but their Fab arms are different due to the heterodimerization of Fc domains, resulting in bispecificity.

The earliest approach to the production of asymmetric IgG-like bsAb was to generate quadromas by the fusion of two hybridomas (Figure 1h) [40]. Because two each of different heavy and light chains are produced in quadroma cells, their random assembly results in 10 unique combinations (16 possible combinations with six identical pairs) of which only one would have the desired bispecificity. Assuming unbiased random assembly of heavy and light chains, only 12.5% of the produced IgG molecules would be bispecific, which needs to be separated from the rest of monospecific or non-functional IgGs. Catumaxomab (Removab^®^) is an EpCAM×CD3 bsAb produced by quadroma technology and was approved by European Medicines Agency (EMA) in 2009 for the treatment of malignant ascites but was withdrawn from the market in 2017. It is a mouse/rat hybrid IgG, with mouse anti-EpCAM IgG2aκ and rat anti-CD3ε IgG2bλ half-antibodies [41]. Because of the preferential pairing between the heavy and light chains of the same species, the amount of functional bsAb is higher than what is expected from random heavy/light chain assembly, and it can be purified by protein A and cation exchange chromatographic steps. Catumaxomab is a trifunctional antibody and binds to EpCAM and CD3 on cancer cells and T cells, respectively, as well as recruiting other immune cells such as macrophages and NK cells through the interaction between Fc and FcγRs. Although neither catumaxomab nor the quadroma technology is currently in active use, they validated the concept of asymmetric bsAb and facilitated the development of a next generation of bsAb formats as discussed below.

The problem of the random pairing of heavy and light chains during the assembly of asymmetric IgG-like bsAbs can be overcome by modifications in CH3 domain, light chain, and/or Fd region. CH3 domains of IgG form strong homodimers, and in the case of human IgG1, the dissociation constant is estimated to be in the sub-picomolar range [42]. Through the engineering of the interdomain interface of CH3, preferential formation of Fc heterodimer (and, hence, asymmetric IgG) can be achieved. Various interdomain interface pairs complementary in shape, contour, charge, hydrophobicity, hydrogen bonding, and/or disulfide bond formation have been reported [43]. Initially, two engineered CH3 pairs with steric complementarity (knobs-into-holes, or KiH) were shown to favor heterodimerization (Figure 1i,j) [44]. Mutations of Thr366 in one CH3 domain to bulkier Tyr (T366Y) and Tyr407 in the other CH3 domain to smaller Thr (Y407T) resulted in the heterodimer formation of up to 92%, and similar heterodimer yield was observed from T366W/Y407A pair. In a subsequent study, Thr366 in one CH3 domain was mutated to Trp, and Thr366, Leu368, and Tyr407 in the other CH3 domain were mutated to Ser, Ala, and Val, respectively [45], while the heterodimeric yield of these constructs was comparable to the T366W/Y407A pair of the earlier study, the stability of the CH3 heterodimer improved significantly, probably owing to the enhanced hydrophobic interaction between the domains. Other heterodimerization strategies, mostly relying on steric complementarity and hydrophobic interactions [46,47,48] but also on charge [49], hydrogen bonding complementarities [50], or an interdomain disulfide bond [51,52], have been shown to yield high levels (typically >90%) of stable heterodimers.

Even with the formation of heavy chain heterodimer, mispairing of light chains can result in non-functional Fabs and reduce the yield of functional bsAb. Attaching scFv or scFab to the N-termini of the heterodimeric Fc domains is one way to prevent the noncognate pairing of heavy and light chains [49,53,54]; however, these formats are different from true IgG due to the presence of linkers and/or the lack of CH1/CL domains in case of scFv. The mispairing problem can also be addressed by a common light chain approach, in which an identical light chain is employed in both Fab arms (Figure 1i). Of the two chains that comprise an antibody, the heavy chain generally makes a greater contribution to antigen binding, and it is often possible to change the whole light chain while maintaining the binding activity of the antibody [55], or to isolate target-specific antibodies from a common light chain antibody library [56]. However, the common light chain approach is not always easily applicable because for many antibodies the light chain also contributes significantly to the antigen binding and finding a light chain that can functionally pair with two different heavy chains is a difficult task. Recently approved hemophilia A drug Hemlibra™ (emicizumab) is an asymmetric IgG-like bsAb employing a common light chain [55].

An elegant solution to the light chain mispairing problem in asymmetric IgG is the CrossMab format. In this method, instead of Fd-LC pairing to produce Fab regions, CH1 and CL in one of the Fab arms of the asymmetric IgG are swapped for each other (CrossMab^CH1-CL^) (Figure 1j) [57]. CrossMabs can be produced in other configurations, such as Fd/LC swap (CrossMab^Fab^) or VH/VL swap (CrossMab^VH-VL^); however, they are prone to side product formation such as nonfunctional monovalent heavy chain heterodimer and Fab in case of CrossMab^Fab^, or IgG-like nonfunctional molecule produced by Bence-Jones V_L_ dimer formation in case of CrossMab^VH-VL^. An Ang-2×VEGF CrossMab^CH1-CL^ based on anti-VEGF bevacizumab and anti-Ang-2 LC06 was produced with 82% yield, along with 4%~7% of various side products lacking one or more of the four chains comprising the complete CrossMab. The affinity of each Fab arm was essentially the same as that of the parental antibody, and the thermal stability was also similar to that of conventional IgG. The bsAb, vanucizumab, in combination with mFOLFOX-6 failed improve the progression-free survival of metastatic colorectal cancer (mCRC) patients relative to the bevacizumab-treated group [58]. A different Ang-2×VEGF CrossMab, faricimab, is being evaluated in a number of phase 3 clinical trials for the treatment of neovascular age-related macular degeneration (nAMD) (NCT03823287 and NCT03823300) and diabetic macular edema (NCT03622580 and NCT03622593). Another sophisticated approach to minimizing LC mispairing is the designed orthogonal Fab interfaces [59,60], which enable >90% correct HC-LC pairing. Electrostatic and steric complementarities in variable (VH:VL) and constant (CH1:CL) domain interfaces, introduced by the mutation of key interface residues, facilitate the cognate Fab pairing; however, the differences in variable domain sequences among antibodies may make it difficult for this approach to be universally applied. Extensive engineering of the CH1:CL domain interface was reported to make the mutant light chains to correctly pair with their cognate mutant heavy chains (up to >99% functional bsAb) without having to introduce mutations to variable domains and thus making the orthogonal Fab interface approach more generally applicable [61].

Instead of introducing all four genes (two for heavy and two for light chains) into host cells as in CrossMab production, asymmetric IgG-like bsAbs can also be produced by controlled Fab arm exchange (cFAE) (Figure 1k) [62,63]. In this method, two monospecific IgGs each with different antigen specificity and harboring a mutually complementary, homodimer-destabilizing mutation in the CH3 domain (e.g., F409L in one IgG and K409R in the other) are produced and purified separately. The two mutant IgGs are mixed in reducing condition (for disulfide bond cleavage) to form asymmetric bsAb via preferential heterodimerization of the mutant CH3s. The method is highly efficient with ≥95% yield of functional bsAb as determined by cation exchange chromatography, hydrophobic interaction chromatography, and mass spectrometry [63]. Two cFAE bsAbs are currently in clinical evaluation: JNJ63709178, a CD123×CD3 bsAb produced by cFAE between F409L/K409R mutant IgGs (“DuoBody”), is in a phase 1 clinical trial for relapsed or refractory acute myeloid leukemia (NCT02715011), while JNJ61186372 (an EGFR×cMet DuoBody) is in a phase I clinical trial for advanced non-small cell lung cancer (NCT04077463 and NCT02609776).

### 2.2. Affinity of bsAbs

As with conventional antibody therapeutics, the affinity of bsAbs is one of the critical determinants of their pharmacological properties. High-affinity binding is usually desirable, although some bsAbs with low to moderate affinities were shown to have exceptionally high potency and efficacy (see below). At the same time, the affinity itself can also be influenced, usually negatively, by the architecture of the bsAb; elaborate engineering is often necessary to restore the decreased affinity. In the following subsections, the influence of bsAb design on its affinities and the engineering approaches to optimize them, as well as the relationship between bsAb affinity and its potency and efficacy are discussed, with various examples of bsAb molecules, their affinities and optimization, and comparison of biological activities provided.

#### 2.2.1. Affinity and bsAb Formats

Apart from the intrinsic dissociation constant of the parental monospecific antibodies, the affinity of each target-binding unit of a bsAb is also affected by its format and structure. For example, an scFv and an Fv with identical variable domains may have different affinities, possibly due to the presence of a peptide linker in scFv and the conformational constraint it imposes: a CD19×CD3 DART (two Fv units) with the identical monospecific units to blinatumomab showed ~two-fold higher affinities to both antigens and stronger cytotoxicity toward CD19+ cell lines than the BiTE molecule (two scFv units) [16]. For multivalent bsAb formats, avidity effect can also enhance the apparent affinity significantly: AFM11, a CD19×CD3 TandAb, exhibited ~eight-fold higher affinities to both targets than the BiTE molecule consisting of identical variable domains [21]. In case of BiTEs, the order of variable domains also had some influence on the binding activity among domain-order variants of blinatumomab. Weaker binding to CD3 was observed for BiTEs with anti-CD3 scFv in V_L_-linker-V_H_ configuration, especially when it is located on the C-terminal side of the molecule (i.e., VL_CD19_-VH_CD19_-VL_CD3_-VH_CD3_), whereas anti-CD19 scFv showed a strong binding to target cells regardless of the location or domain order [64]. Similar effects of molecular architecture on the affinity are likely to be observed in other fragment-based or appended IgG bsAbs, although the preferred configurations would vary widely among different formats and monospecific units.

Steric hindrance caused by the attachment of additional binding units is also a factor influencing the affinity of bsAbs. A study on the construction and characterization of various formats of CEA×DOTA IgG-scFvs as well as DVD-Ig [65] showed that the affinity of anti-CEA Fv on IgG portion of the molecule was largely unaffected by the fusion to scFv, regardless of the format, whereas the affinity of the attached anti-DOTA scFv could vary >10-folds depending on the site of attachment. The anti-DOTA affinity was highest when the scFv was fused to the C-termini of the light chains (13 nM), and lowest in DVD-Ig format (213 nM, anti-DOTA Fv in proximal position). These characteristics cannot be generalized though; for example, the affinities of various anti-EGFR/anti-IGF-1R IgG-scFv bsAb constructs toward both antigens were all comparable to one another and to the parental antibodies regardless of the site of attachment [40]. Linker flexibility, size and structure of the target antigen, epitope location, and the orientation of binding interaction are all likely to contribute to the binding characteristics of the appended or inserted binding units of a bsAb. A systematic study of DVD-Igs with linkers of various sizes and sequences revealed that (1) the affinity of the proximal Fv tended to be higher with longer linkers, (2) linkers based on CH1-CH2 hinge sequence of human IgG1 performed better than those based on natural V-C junction sequences or polyglycine, and (3) partial or full cleavage of the distal Fv largely restored the binding activity of the proximal Fv [36]. In a later study, a series of DVD-Ig proteins with an identical anti-VEGF proximal Fv, several different distal Fvs against another target, and V-C junction-based linkers of different lengths connecting them were constructed and their binding kinetics were measured [37]. A > 20-fold reduction in affinity was observed for the anti-VEGF proximal Fv when short linkers (5–6 amino acids) were used instead of long ones (12–13 amino acids). Moreover, the affinity of the proximal Fv was 2.5–5 times higher when a short and a long linker were used for heavy and light chains, respectively (short/long), than the other (i.e., long/short) configuration. Moreover, the binding affinity of the proximal Fv varied by >three-fold when different distal Fvs were employed in otherwise identical DVD-Ig proteins. These results suggest that simultaneous engineering of multiple components is needed in optimizing these types of bsAbs and the optimal bsAb design, even within a same format, varies widely among different specificities.

The affinities of the parental antibodies are not likely to change significantly in the asymmetric Fc-based bsAbs, which have an essentially identical structure to those of native IgGs. One possible exception is the CrossMab format, which has unnatural VH-CL and VL-CH1 linkages not found in conventional immunoglobulins. The effect of the domain swapping on the affinity of CrossMab^CH1-CL^, as in the cases of other bsAb formats, varies among different variable domains: While the affinity of the CH1-CL-crossed anti-VEGF Fab arm of vanucizumab (an Ang-2×VEGF CrossMab) is essentially identical to that of its parent bevacizumab with *K*_D_ < 0.1 nM [57], the affinity of faricimab (another Ang-2×VEGF CrossMab with a crossed anti-Ang-2 Fab arm) toward Ang-2 was ~five-fold lower than that of the parental anti-Ang-2 mAb LC10 (22 nM and 4.1 nM, respectively) [66]. The differing effects of the CrossMab domain crossover on their affinities once again highlight the technical difficulties associated with optimizing the affinities of bsAbs, and the necessity to further develop more advanced technological platforms in the field of bsAb engineering in order to efficiently generate therapeutic candidates of desired qualities.

#### 2.2.2. Effects of Affinity on the Biological Activity of bsAbs

The effects of bsAb affinity on their biological activities can be best assessed by comparing the biological activities of different bsAbs targeting a same pair of antigens. CD19 and CD3 are an ideal pair for such analyses: companies and researchers have developed and reported many CD19×CD3 bsAbs of different formats and affinities, and their biological activities were studied in great detail, often in a comparative fashion. At least three of them (blinatumomab, AFM11, and duvortuxizumab) have been tested in clinical studies; they are of different formats and based on different parental antibodies with different affinities, thus providing a suitable set of data for analyzing the effect of bsAb format and affinity on their functions.

The anti-CD19 moiety of AFM11 is a humanized affinity-matured version of the clone HD37 [22], the parental antibody used for the construction of blinatumomab [64], while duvortuxizumab (a DART-Fc) has humanized anti-CD19 mAb BU12 as its targeting moiety [67]. The affinities of blinatumomab, AFM11, and duvortuxizumab for human CD19 are 2.1, 0.37, and 2.0 nM, respectively. The anti-CD3 Fv of blinatumomab is from the clone TR66, while humanized-affinity matured mAb UCHT and humanized mAb XR32 were used for AFM11 and duvortuxizumab, respectively. Unlike anti-CD19 Fvs whose affinities differ from one another by less than an order of magnitude, the anti-CD3 affinities of these bsAbs vary more widely, with dissociation constants of 120 nM, 2.1 nM, and 21 nM for blinatumomab, AFM11, and duvortuxizumab, respectively. Therefore, it is of interest to analyze whether the difference in anti-CD3 affinities translates into differences in the biological activities of these bsAbs. Several head-to-head in vitro and in vivo studies comparing blinatumomab, a clinically approved bsAb, with other CD19×CD3 bsAbs have been reported. Because of the differences in study details, including cell lines used, incubation condition, and protein preparation, direct comparison of the results from different studies is difficult; nonetheless, they provide valuable insights into the mechanism of action of these highly potent anticancer drugs.

AFM11 is much more potent than blinatumomab in vitro, with sub-picomolar EC_50_ when targeting CD19 + MEC-1 cells or NALM6 cells in the presence of primary human T cells. At effector: target ratio (E/T) of 25, AFM11 exhibited sub-picomolar EC_50_ after 1 h incubation, whereas blinatumomab showed EC_50_ > 20 pM under the same condition. Upon 23 h incubation the two bsAbs showed comparable potency (EC_50_) at E/T = 5 against NALM6 cell line, however AFM11 maintained high levels of potency and efficacy at lower E/T. Even at E/T ratio of 0.2 AFM11 induced 60% lysis of target cells versus 20% lysis for blinatumomab, indicative of the robust serial killing activity with which the TandAb functions catalytically and a single AFM11 molecule can participate successively in the killing of multiple target cells. The efficacies (maximal % lysis) of AFM11 and blinatumomab were comparable at E/T > 2; however, a higher concentration was required for the latter to achieve the maximal lysis. Duvortuxizumab was also shown to be more potent than blinatumomab, and EC_50_ against Raji/GF cell line using human T lymphocytes at E:T ratio of 10:1 was 0.17 pM (0.019 ng/mL), compared with 7 pM (0.38 ng/mL) for blinatumomab after 24 h incubation [67]. While the potency of these bsAbs varies rather widely, it should be noted that the affinity is not likely to be the only contributor to these differences in potency; differences in the structures and epitopes of these bsAbs may play roles by, e.g., forming tighter immune synapse [16].

It is noteworthy that the affinity of blinatumomab for CD3 is fairly low with *K*_D_ ≈ 100 nM [68], but its EC_50_ toward CD19-expressing cancer cells are in the picomolar range [12,68]. Because of the high affinity for CD19 (*K*_D_ = 2.1 nM), multiple molecules of blinatumomab are bound to the target cell surface, through which robust immune synapses between the target and effector cells can be formed by the avidity effect despite the low affinity of blinatumomab for CD3. On the other hand, a CD19×CD3 TandAb with sub-nanomolar affinity for CD3 was reported to have EC_50_ in the nanomolar range toward the same cancer cell lines [12]. It has initially been suggested that the low affinity of blinatumomab for CD3 enables efficient detachment of T cells from target cells after cell death, allowing them to serially kill other target cells and effectively lowering EC_50_ [69]: at E/T = 0.1, 50% of NALM6 cells were lysed after 24 h incubation in the presence of 1 ng/mL blinatumomab. This explanation needs to be interpreted with caution, though, firstly because high potency (low EC_50_) and serial killing activity (low E/T) are not necessarily correlated, and secondly because CD19×CD3 bsAbs with a higher affinity for CD3 such as AFM11 could still induce serial killing of target cells, and actually shows greater potency than blinatumomab (see above). In addition, when a high-affinity (low nanomolar *K*_D_ value) anti-CD3 scFv was employed in making an anti-BCMA BiTE, cytotoxic activity with subpicomolar to picomolar EC_50_ values could still be observed against human multiple myeloma cell lines [70]. In contrast to the initial TandAb construct based on the anti-CD3 clone OKT3 [21], the subsequently developed T-cell engaging bsAbs utilized different anti-CD3 moieties with presumably different epitopes on CD3ε and repeated proof of picomolar potency as a part of T-cell engaging bsAbs [71], which may better explain the differences in potency of these molecules than the anti-CD3 affinity alone does.

## 3. Antibody–Drug Conjugates

Antibody–drug conjugates (ADCs) are another class of highly potent antibody-based therapeutics. ADCs consist of three integral components: (1) an antibody targeting cancer cell-specific antigen, (2) a cytotoxic payload, and (3) a chemical linker that connects the drug and the antibody. Following the binding to cancer cell-surface antigen, ADC is internalized by receptor-mediated endocytosis, and the payload is released by the degradation of the linker or the antibody in the endolysosomal compartment. In developing ADCs, therefore, factors such as target antigen biology, specificity of the antibody, cytotoxicity and mechanism of action of the payload drug, the stability and cleavage of the linker, and the sites of linker attachment all need to be carefully considered.

### 3.1. Target Antigens

Ideally, the target antigen for ADC needs to be overexpressed on cancer cell surfaces with no or negligible expression on normal healthy tissues. Because the main role of the antibody in ADC is to deliver the cytotoxic payload to cancer cells, the biological functions of the target antigen or their inhibition by the antibody are considered less important than for conventional therapeutic antibodies (but see below). Instead, the antigen needs to be endocytosed upon antibody binding, in order to deliver the payload into cancer cells. The level of surface expression is also crucial for ADC target antigen since the number of target molecules per cell, which ranges from less than a thousand to over a million, is an important determinant of the payload delivery efficiency. A number of cancer targets meet these criteria, and ADCs targeting these molecules have been approved or are in various stages of clinical development. Many of the anti-cancer ADCs target hematological cancers; these cancers are better accessible than solid tumors by large molecules, and normal as well as cancerous cells of a specific immune cell subset can be targeted and depleted without excessive toxicity. However, solid tumors constitute ~90% of all cancer incidences [72], and there are also considerable efforts to develop ADCs against solid tumor targets.

Hematological cancer targets for approved ADCs include CD33, CD30, CD22, and CD79b. These antigens typically are restricted to a specific immune cell lineage, and on-target toxicities to normal tissues and organs can be minimized. CD33 is one of the earlier ADC markers targeted by gemtuzumab ozogamicin (Mylotarg™) for the treatment of acute myeloid leukemia (AML). Its expression is restricted to the cells of myeloid lineage, with 90% of AML cases expressing the antigen on >20% of the leukemic blasts [73]. CD33 is internalized following antibody binding [74], making possible the development of ADC targeting this antigen. However, the expression of CD33 on normal myeloid cells, as well as hepatic sinusoidal endothelial cells [75] and Kupffer cells [76] which can be derived from bone marrow progenitors, causes target-dependent adverse events such as myelosuppression and hepatic toxicity [77]. Partly due to the toxicity, Mylotarg was withdrawn from the U.S. market in 2010 and re-approved by the FDA in 2017 for the treatment of CD33+ AML with a lower recommended dose and a revised treatment schedule [78]. Another ADC approved for hematological cancers, brentuximab vedotin (Adcetris™) targets CD30, a member of tumor necrosis factor receptor superfamily (TNFRSF) expressed on activated B cells, T cells, and NK cells, as well as a number of T- and B-cell malignancies, including Hodgkin lymphoma and anaplastic large cell lymphoma. While its role in lymphocyte proliferation and differentiation is inconclusive or contradictory [79], CD30 is a suitable target for ADC development thanks to the restrict expression pattern and rapid internalization upon antibody binding, and adverse events are usually low-grade and manageable for Adcetris™ [80]. The examples of CD33 and CD30 suggest that the lineage-specific expression profile and the efficient internalization are two of the key factors determining the successful ADC targets for hematological cancers.

CD22 and CD79b are markers of B-cell lineage and are targeted by inotuzumab ozogamicin (Besponsa™) and polatuzumab vedotin (Polivy™), respectively. Both are restricted to lymphocytes of B-cell lineage: CD22 is most prominently expressed on mature B cells while CD79b (a B cell receptor complex component) is a pan B-cell marker and expressed in virtually all immature and mature B cells [81]. Because the loss of B-cell targets such as CD19 is a major mechanism of treatment resistance, these novel targets are expected to provide new therapeutic options for, e.g., anti-CD19-refractory B-cell malignancies [82]. Moreover, the inherent biological function of CD79b as a component of B-cell receptor (BCR) complex enables the efficient internalization and delivery of the bound ADC to the lysosomes, resulting in highly potent cancer cell killing [83,84]. On the other hand, while CD19 is a prototypical target of B-cell cancers for T cell engaging bsAbs (see above) or CAR-T cells [85] and coltuximab ravtansine (SAR3419, an anti-CD19 ADC) has also been evaluated in clinical trials (NCT01472887) [86], the antigen was found to internalize only in CD21-negative cells [87], exemplifying a technical hurdle of antigen internalization associated with the target selection for ADC.

Most cell-surface targets of solid tumors are also expressed on normal tissues, albeit in lower abundance, which makes it more difficult to simultaneously achieve efficacy and safety of ADCs for these malignancies. HER2 is the most successful ADC target for solid tumors to date, and two ADC drugs targeting it have been approved: trastuzumab emtansine (T-DM1, Kadcyla™) and trastuzumab deruxtecan (DS8201a, Enhertu™). About 15% of breast cancers were found to be HER2+ [88] along with other solid tumors such as gastric cancer [89], and targeted therapy against HER2 proved efficacious using kinase inhibitors, monoclonal antibodies, and ADCs [90]. HER2 expression level in breast cancers overall are only <2-fold higher than in normal breast tissues and the normal expression level of HER2 is higher than the expression levels on cancer cells of some of other ADC targets [83], which would make HER2 an unattractive target for ADC development. However, HER2 is highly overexpressed in a subset of breast cancers (>10^6^ molecules/cell) [91], and ADCs with optimal combination of targeting antibody, linker, and payload can selectively kill HER2-overexpressing cancer cells while minimizing overt toxicity [92]. In addition, HER2 is an oncogenic driver for many HER2+ cancers, which makes it less likely for these cancer cells to become refractory to anti-HER2 ADCs by downregulating the antigen [83], a major mechanism of resistance to ADCs targeting antigens with no apparent biological functions for cancer cell survival. The success of anti-HER2 ADCs demonstrates that an antigen with broad normal tissue distribution may be targeted by ADCs through proper understanding of target biology, patient stratification, and optimized design and a combination of ADC components.

Another solid tumor target, nectin-4 is a calcium-independent cell adhesion molecule overexpressed in bladder and other cancers, and the target for the recently approved enfortumab vedotin (AGS-22M6E, Padcev™) [93]. It is hypothesized that nectin-4 plays an important role in the formation and maintenance of adherens junctions and in the establishment of apico-basal cell polarity [94]. Nectin-4 is also broadly expressed in many normal epithelial tissues in low-to-moderate levels; however, its expression levels in cancers are much higher [93], resulting in an acceptable safety profile and therapeutic window for enfortumab vedotin [95]. Although nectin-4 forms homo- and heterodimers with other nectins and the parent antibody for AGS-22M6E (enfortumab) can inhibit the interaction between nectin-4 and nectin-1 by binding to the V domain of nectin-4, the unconjugated antibody did not show any effect on cell viability [93]. On the other hand, the vcMMAE-conjugated antibody potently induced cancer cell death with IC_50_ of 0.25 nM (37.8 ng/mL) for T47D cells, implying efficient internalization of the ADC upon the binding to nectin-4.

ADCs targeting other solid tumor markers such as mesothelin and EGFR are also under clinical development (see below in the cytotoxic drug subsection for the additional information about these ADCs) [96]. Mesothelin is highly expressed in mesothelioma and adenocarcinomas of ovary, pancreas, and lung, with low, limited normal tissue distribution, and internalizes efficiently upon antibody binding [97]. Anetumab ravtansine, an anti-mesothelin ADC, is under phase 2 clinical development. Depatuxizumab mafodotin is an anti-EGFR ADC targeting a tumor-selective cryptic epitope on the CR1 domain of EGFR [98]. Probably as a result of the tumor selectivity, depatuxizumab mafodotin did not cause dose-limiting dermatological adverse events or diarrhea characteristic of other anti-EGFR therapies, even though EGFR is broadly expressed in normal tissues [99]. Similar to the cases of the anti-HER2 ADCs, the development of the anti-EGFR ADC suggests that the technical hurdles associated with ADC targets may be overcome by taking advantage of the target biology in the context of cancer cells.

### 3.2. Cytotoxic Drugs

**Figure 2:**
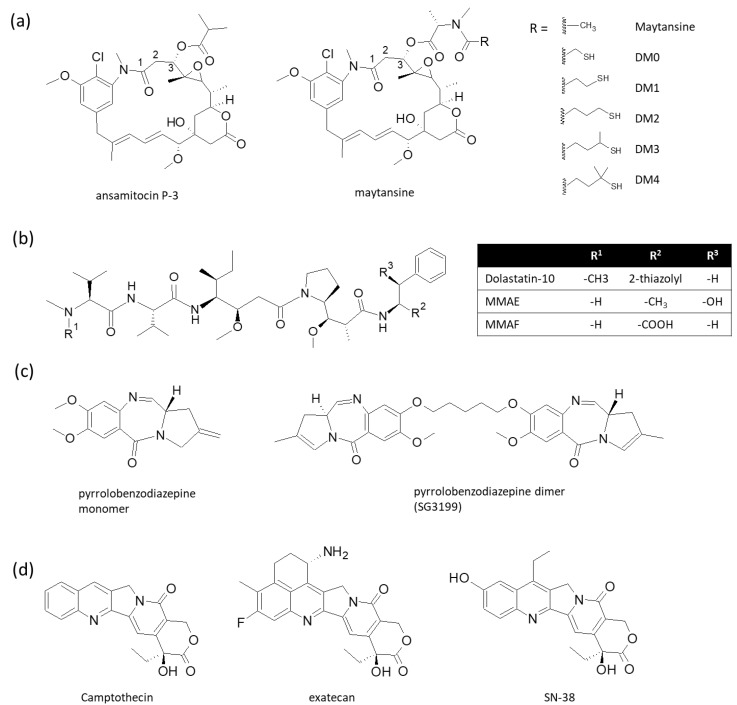
Structures of commonly used antibody–drug conjugate (ADC) payloads. (**a**) Structures of maytansinoids and their semisynthetic precursor, ansamitocin P-3. (**b**) Structures of auristatin. “Monomethyl” (“MM” of MMAE and MMAF) refers to the methylation status of the N-terminal amino group, which is dimethylated in the natural compound dolastatin 10. (**c**) Structures of PBD monomer and a dimer (SG3199). (**d**) Structures of camptothecin and its derivatives exatecan and SN-38.

The cytotoxic drugs for ADC need to be highly potent, considering the limited amount of payload molecules per cell that can be delivered by ADC to induce cancer cell death [100]. Tubulin inhibitors such as auristatins and maytansinoids are commonly used as ADC payloads. These molecules are too toxic to be used by themselves as therapeutic drugs; however, when conjugated to cancer-targeting antibodies, they can effectively kill cancer cells at a very low concentration. For example, auristatin E shows an average IC_50_ of 3.2 ± 0.51 nM against a panel of 39 human cancer cell lines upon 1 h exposure [101], compared with an average IC_50_ of 166 nM for vinblastine (another tubulin inhibitor) or 631 nM for doxorubicin. Another class of ADC payload is molecules that target DNA, including duocarmycin (DNA alkylation), calicheamicin (DNA double strand cleavage), camptothecin analogues (topoisomerase inhibitor) such as SN-38 and exatecan, or pyrrolobenzodiazepine (PBD) dimers (DNA strand crosslinking) [102]. A majority of the ADC candidates currently under clinical evaluation employ one of the three major classes of cytotoxic drugs, namely maytansinoids, auristatins, and PBD dimers (Figure 2) [96], but other classes of payloads, such as calicheamicin (for gemtuzumab ozogamicin and inotuzumab ozogamicin), duocarmycin, SN-38 [102], or exatecan [103] are also used.

Maytansine is a macrolide natural product isolated from the *Maytenus* species of plants [104] and inhibits the longitudinal tubulin interactions in microtubules by binding to the rhizoxin binding site on tubulin [105]. Upon 72 h of exposure, maytansine derivatives S-methyl-DM1 and S-methyl-DM4 induced the killing of KB cells (identical to HeLa cervical adenocarcinoma cell line) with IC_50_ values of 22 and 26 pM, respectively [106]. Maytansinoids share the identical macrolide ring structure of maytansine, and differ from one another in the substituent at C3 (Figure 2a) [107]. The total synthesis of maytansine has been reported [108], but commercially maytansinoids are prepared semi-synthetically from ansamitocin P-3 which can be produced by microbial fermentation [109,110]. Ansamitocin P-3 shares the same maytansine macrolide ring structure and after a reduction of C3 ester and re-esterification, can be converted to other maytansinoids, including maytansine and derivatives of maytansine known as DM0-DM4 [111]. DM compounds retain the potent cytotoxic activity of maytansine and have a thiol group for the conjugation to antibodies via a linker. As well as trastuzumab emtansine, which uses DM1 as its cytotoxic payload and has been approved for the treatment of HER2-positive breast cancer, a number of maytansinoid-conjugated ADCs are under clinical evaluation, including naratuximab emtansine (Debio 1562, an anti-CD37 ADC with DM1, phase 2; NCT02564744), mirvetuximab soravtansine (an anti-FOLR1 ADC with DM4, phase 3; NCT02631876), and anetumab ravtansine (an anti-mesothelin ADC with DM4, phase 2; NCT03455556, NCT03126630, NCT03926143, NCT03023722, NCT03587311).

Auristatins are another class of tubulin inhibitors that bind to the vinca site on tubulin and induce curvature and longitudinal polymerization of tubulin dimers [112,113]. They also inhibit the nucleotide exchange of tubulin [114] and have been suggested to interfere with microtubule formation by stabilizing extended M-loop conformation [113]. Auristatins are synthetic derivatives of dolastatin 10, a peptide natural compound from the sea hare *Dolabella auricularia* [115]. The most commonly employed auristatins in ADC are monomethyl auristatin E and F (MMAE and MMAF), which differ from dolastatin 10 by N-terminal methylation (*N*-dimethyl for dolastatin 10 and *N*-monomethyl for MMAE and MMAF) and the residue at the C-terminus (norepinephrine for MMAE and phenylalanine for MMAF) (Figure 2b). Due to the presence of a negative charge at the C-terminus, MMAF is less membrane-permeable than MMAE. As a result, MMAF has a higher IC_50_ value in vitro and is less capable of mediating the bystander killing effect [100]. However, the additional negative charge of the carboxy-terminus of MMAF interacts with Arg278 of tubulin, which is probably the reason for the higher affinity of MMAF for tubulin [113]. Brentuximab vedotin (Adcetris™), polatuzumab vedotin (Polivy™), and enfortumab vedotin (Padcev™) are approved ADCs with MMAE as a payload. Several other ADCs with MMAE or MMAF payloads are in clinical trials; these include depatuxizumab mafodotin (an anti-EGFR ADC with MMAF, phase 3; NCT03419403, NCT02573324) and AGS-16C3F (anti-CD203c ADC with MMAF, phase 2; NCT02639182).

PBDs are a class of antitumor/antibiotic natural compounds produced by actinomycetes [116] and capable of selectively binding to the minor groove of the DNA double helix at a 5′-(A/G)G(A/G)-3′ sequence to form a covalent bond to the amino group of guanine base [117]. Synthetically produced PBD dimers exhibit potent cytotoxicity by crosslinking DNA strands, and a PBD dimer, SG3199 (Figure 2c), could inhibit the growth of various human cancer cell lines with subnanomolar GI_50_ (drug concentration at 50% growth inhibition) [118]. The cytotoxicity of PBD dimer depends on the structure of PBD core and exocyclic substituents, as well as the structure and length of the dimerization linker [119]. Rovalpituzumab tesirine (Rova-T; an anti-DLL3 ADC) is an example of an ADC with PBD dimer payload; once internalized, the linker of its tesirine payload is cleaved and the cytotoxic drug SG3199 is released into the cytosol [118]. Although Rova-T was withdrawn from phase 3 trials due to the lack of survival benefit, many PBD dimer-conjugated ADCs are in earlier phases of clinical trials to exploit the exceptionally potent cytotoxicity of this class of payload molecules [119].

The recently approved trastuzumab deruxtecan (Enhertu™) employs exatecan, a synthetic derivative of the topoisomerase I inhibitor camptothecin [103], as a payload (Figure 2d). Another camptothecin derivative, SN-38, is an active metabolite of anticancer drug irinotecan and used in e.g., sacituzumab govitecan [120]. Camptothecin is an alkaloid isolated from the plant *Camptotheca acuminate* and potently inhibits the growth of cancer cells with nanomolar IC_50_ [121]. Exatecan was synthesized by adding a six-membered ring between the rings A and B of camptothecin, and a fluorine to the ring A, to make it more soluble in water. It is on average 6–7 times more potent than camptothecin or SN-38 with sub-nanomolar GI_50_ for a majority of cancer cell lines tested and inhibits topoisomerase I with IC_50_ = 0.975 μg/mL compared with 2.71 μg/mL for SN-38 and 23.5 μg/mL for camptothecin [121].

PBD dimers are the most potent class of cytotoxic drugs among commonly used ADC payloads, followed by maytansinoids, auristatins, and calicheamicin showing comparable potency to one another [122]. However, cytotoxicity is not the only criterion for the selection of the payload for ADC construction, as exemplified by the clinical success of trastuzumab deruxtecan whose exatecan payload arguably has a weaker cytotoxicity than maytansinoids but which as an ADC is more potent than trastuzumab emtansine [122], or the employment of MMAF in some auristatin-based ADCs despite its lower in vitro potency than MMAE (see above). Along with other ADC components, including the conjugation chemistry and the linker structure, the chemical and biological properties of the payload may also influence the clinical outcome of ADC therapies. For example, the bystander killing effect, mediated by the drugs released from a dying target cell entering adjacent cells, varies among the payload molecules with different charges and/or hydrophobicity, and can have a significant effect on the potency of an ADC [123]. The hydrophobicity, in turn, may also affect the drug-antibody ratio (DAR) and the solubility of the ADC. Therefore the ideal payload for an ADC should be determined by considering its compatibility with other ADC components as well as the biology of the targeted cancer: For example, bystander killing may be more important for a cancer with heterogeneous expression of the target antigen in which target-overexpressing cells are surrounded by target-negative cells [123].

Resistance to the ADC treatment is another factor that is related to the payload selection. Cancer cells may acquire the overexpression of ABC transporters for the efflux of chemotherapeutic drugs, most notably P-glycoprotein 1 (Pgp, also known as ABCB1, MDR1 or multidrug resistance 1), and many ADC payload molecules are substrates for these transporters that have a broad substrate specificity for hydrophobic molecules [124]. For example, the acquired resistance of HL-60 cells chronically exposed to gemtuzumab ozogamicin was attributed the induction of overexpression of MDR1 [125]. Other common ADC payloads such as MMAE [126] and maytansinoids [107] are also substrates for MDR1 and induce upregulation of drug transporters in cancer cells upon ADC treatment. In contrast, PBD dimers [118] and exatecan [127] are not efficiently pumped out by this mechanism, which may in part explain the sensitivity of T-DM1-resistant cancer cells to trastuzumab deruxtecan [128] and the increased interest in these classes of ADC payloads.

### 3.3. Linkers

**Figure 3:**
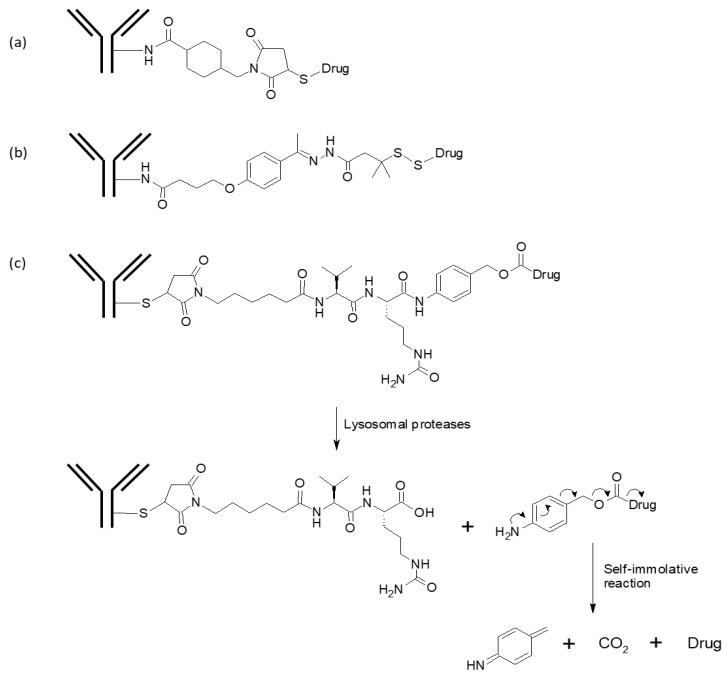
Representative structures of different types of ADC linkers. (**a**) Non-cleavable thioether linker of trastuzumab emtansine. (**b**) Acid-labile hydrazone linker of gemtuzumab ozogamicin and inotuzumab ozogamicin, with an additional disulfide linkage. (**c**) Enzyme-cleavable linker of brentuximab vedotin with Val-Cit motif.

Linkers for ADCs can be classified into cleavable and non-cleavable ones. Non-cleavable linkers, e.g., the one in trastuzumab emtansine (Figure 3a), remain attached to the cytotoxic drug after proteolytic degradation of the antibody in the lysosome. The drug, with an attached linker and an amino acid or short proteolytic peptide to which the linker is conjugated, is released to the cytosol and exerts its cytotoxic activity [129,130]. Cleavable linkers, on the other hand, are cleaved by lysosomal enzymes, or by low pH or a reducing environment inside endosomes; the drug-attached fragment after cleavage is typically removed by self-immolative reaction, releasing the free cytotoxic payload [131]. Brentuximab vedotin, for example, has a cathepsin-cleavable linker (valine-citrulline linker), and MMAE is released by the self-immolative reaction of the remaining linker fragment [101].

Regardless of the type, the linker for ADC has to be highly stable in blood in order to minimize the release of highly cytotoxic free drugs into circulation and to maintain maximum amount of intact ADC molecules targeting cancer cells. Gemtuzumab ozogamicin (Mylotarg™) and inotuzumab ozogamicin (Besponsa™), for example, both have an acid-labile hydrazone linker, which is relatively stable at pH 7.4 but hydrolyzed with high efficiency in the acidic environment of lysosome [74]. However, the acid-labile hydrazone linker can be considerably less stable than some of the other types of cleavable linkers, which may contribute to the narrow therapeutic window and high toxicity of gemtuzumab ozogamicin [132]. Another type of non-enzymatic cleavable linker is the disulfide linker, which is stable in bloodstream but reduced to free thiols by a high intracellular concentration of reduced glutathione. Upon internalization, the drug-linker is released to cytosol after antibody degradation or linker cleavage in lysosome, and the disulfide bond in the linker is reduced by cytosolic glutathione. Anetumab ravtansine [97] and mirvetuximab soravtansine [133] (see above) are examples of ADCs with disulfide linkers, as well as gemtuzumab ozogamicin and inotuzumab ozogamicin whose linker contains not only the hydrazone moiety but also a disulfide linkage (Figure 3b) [134]. The serum stability of disulfide linkers depends on the substitution on the α-carbon atoms of the disulfide linkage [130]. Linkers with an unsubstituted disulfide bond were readily cleaved in circulation, and ADCs with such linkers showed a higher toxicity and a lower efficacy. On the other hand, ADCs with highly substituted disulfide linkers (e.g., Tmab-SSNPP-DM4 in [130]) had comparable stability to the ADC with a non-cleavable linker, with up to ~80% of the ADC retaining its conjugated drugs in vivo after seven days.

Enzymatically cleavable linkers are cleaved by lysosomal enzymes, most notably cathepsin B, which is overexpressed in many cancers [135]. Cathepsin B is a lysosomal cysteine protease with carboxydipeptidase and endopeptidase activities at acidic and neutral pH, respectively; as a carboxydipeptidase, it cleaves the penultimate peptide bond, preferentially after a basic amino acid preceded by an aliphatic or aromatic amino acid (e.g., Phe-Arg) [136]. Chemical linkers with a suitable dipeptide motif are also cleavable by the carboxydipeptidase activity of cathepsin B, among which the valine-citrulline (Val-Cit) motif is most commonly used (Figure 3c) [137]. Brentuximab vedotin, polatuzumab vedotin, enfortumab vedotin, and glembatumumab vedotin (an anti-gpNMB ADC, phase 3; NCT01997333) are some of the ADCs that use the Val-Cit linker to conjugate MMAE to the antibodies. Another dipeptidyl motif valine-alanine is employed in, e.g., vadastuximab talirine (an anti-CD33 ADC with PBD dimer payload) and rovalpituzumab tesirine. The Val-Ala linker is less hydrophobic than Val-Cit and can achieve a higher drug-to-antibody ratio (DAR) without inducing aggregation [138]. Other than linkers with a dipeptide motif, cathepsin-cleavable Gly-Gly-Phe-Gly (GGFG) tetrapeptide motif has been employed in trastuzumab deruxtecan [139]. The GGFG linker is conjugated to the hydroxyl group of exatecan payload (a topoisomerase I inhibitor) via self-immolative aminomethylene linker. The ADC with a less hydrophobic GGFG linker was stable at a high DAR of 8, resulting in a better antitumor efficacy than trastuzumab emtansine (DAR ~3.5).

While it has been generally believed that cathepsin B is primarily involved in the cleavage of Val-Cit or other dipeptide linkers, recent publications have suggested that other lysosomal enzymes, such as cathepsins S, X, L, or D may also be responsible for the release of the payload [137,140,141]. Moreover, while the Val-Cit linker is highly stable in human plasma, it is much less stable in mouse plasma [101], which makes it difficult to perform and analyze nonclinical PK/PD studies in rodent models. It was later elucidated that mouse carboxylesterase 1C is responsible for the cleavage of the Val-Cit linker in mouse plasma [142]. Efforts to modify the Val-Cit linker to suppress these non-cathepsin B-catalyzed cleavages have resulted in optimized linker designs that potentially have an enhanced stability and a better tumor specificity [141,142,143].

Linkers that are cleaved by non-proteolytic enzymes, including glycosidases [144,145,146] and phosphatases [147,148], are generally in earlier stages of development. Lysosomal glycosidases such as β-glucuronidase and β-galactosidase are often overexpressed in cancers and cleave the glycosidic linkages of β-glucuronide and β-galactoside, respectively. Because these carbohydrates are highly polar and hydrophilic, higher DAR could be achieved with glycosidase-cleavable linkers without significant aggregation of ADC [144], which, in turn, resulted in improved pharmacokinetics and therapeutic index [149]. Lysosomal acid phosphatase and acid pyrophosphatase can cleave linkers with phosphomonoester and phosphoanhydride bonds, respectively [131,148]. Linkers with phosphate or pyrophosphate groups are highly hydrophilic due to the negative charges on the phosphate, helping ADCs remain soluble and monomeric (not aggregated) with hydrophobic payload molecules. Pyrophosphate linkers remained stable in human plasma for seven days, demonstrating their utility in the development of ADCs [148].

### 3.4. Conjugation of Linker-Payload to Antibody

**Figure 4:**
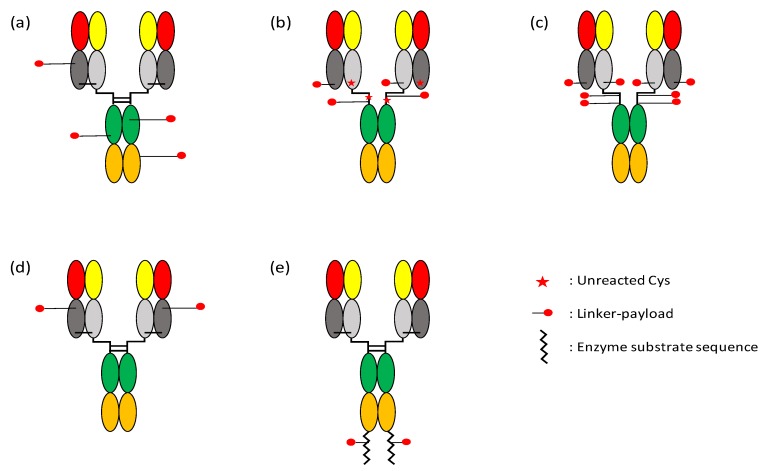
Graphics of representative ADCs with different conjugation methods. (**a**) Random conjugation to primary amines, with hypothetical drug-to-antibody ratio (DAR) = 4. (**b**) Cysteine conjugation after the reduction of interchain disulfide bonds, with hypothetical DAR = 4. (**c**) Complete cysteine conjugation with DAR = 8 (e.g., trastuzumab deruxtecan). (**d**) Site-specific conjugation by the introduction of e.g., cysteines or non-natural amino acids, DAR = 2. (**e**) Site-specific enzymatic conjugation to the introduced substrate sequence, DAR = 2.

In most cases, linker-payload moieties are conjugated to antibodies at lysine or cysteine residues, exposed on the surface either as a part of the native antibody sequence or by point mutations introduced at empirically determined positions. Linker-payloads can be attached to the primary amine of lysine side chain using well-established chemical reagents such as succinimidyl-4-(*N*-maleimidomethyl)cyclohexane-1-carboxylate (SMCC) [150] or *N*-hydroxysuccinimide (NHS)/1-(3-Dimethylaminopropyl)-3-ethylcarbodiimide(EDC) [151]. In trastuzumab emtansine, for example, the semi-stable *N*-hydroxysuccinimide (NHS) ester of SMCC reacts with the antibody’s primary amine to produce an amide bond, while the maleimide moiety reacts with the sulfhydryl group of DM1 to form a stable, non-cleavable thioether linkage (Figure 3a). Typical IgG molecules have on average about 90 lysine residues, among which ~30 have been suggested to be able to participate in conjugation reaction [152]. This may be an underestimation, though, since it has been reported that 70 of 92 primary amines (88 lysine residues and four N-termini) were modified by linker-payload conjugation in trastuzumab emtansine [153]. The large number of modifiable lysines results in a high level of heterogeneity in lysine-conjugated ADCs, both in the number of drug molecules attached per antibody and in the site of linker-payload attachment (Figure 4a). The conjugation site heterogeneity can negatively influence the in vivo stability, efficacy, and pharmacokinetics of ADC [154,155], and recently developed ADCs tend to avoid payload conjugation to lysine residues.

Immunoglobulins have multiple interchain disulfide bonds that can be reduced to free sulfhydryls for conjugation to linker-payload. Unlike lysines, there is no free cysteine residue in constant domains and the framework regions of variable domains of human IgG molecules, and the occurrence of cysteines in complementarity determining regions (CDRs) is relatively uncommon [156]. As a result, cysteine-conjugated ADCs of IgG1 or IgG4 subclass can have maximum DAR of 8 (although for most of them DAR is limited to ~4 for optimal physicochemical properties of the ADC), and are much less heterogeneous than lysine-conjugated ADCs (Figure 4b,c). Brentuximab vedotin, trastuzumab deruxtecan, polatuzumab vedotin, rovalpituzumab tesirine, and many other ADCs are conjugated to drugs via cysteine residues. The linker-payloads are, in most cases, conjugated by maleimide chemistry, although the reaction is highly efficient and cysteine-specific, the thiosuccinimide linkage is somewhat unstable in plasma and undergoes maleimide exchange with free thiols of albumin, cysteine, and glutathione [157], which react with the maleimide liberated from ADC by a retro-Michael reaction [158,159]. Introducing a primary amino group near the thiosuccinimide ring has been reported to alleviate this problem by intramolecularly catalyzed hydrolysis of the ring [160]. The local environment of the conjugation site is also an important factor for the stability of the linkage; a less solvent-exposed, more basic environment promoted the ring-opening hydrolysis of thiosuccinimide, making it resistant to maleimide exchange [157].

The utilization of native lysines or cysteines inevitably results in high levels of ADC heterogeneity, as described above. The introduction of non-native cysteine residues (or other reactive, unnatural amino acids) at solvent-accessible positions for payload conjugation has been pursued to produce highly homogeneous ADCs with more predictable and reproducible DAR (Figure 4d). Thiomab™ technology, for example, enables site-specific payload conjugation with highly homogeneous DAR distribution. In order to identify optimal positions for the introduction of a reactive thiol, every Ser, Ala, or Val residue in the constant domains of trastuzumab Fab had been individually mutated to cysteine [155,161]. Among the mutants (Thiomabs), the A114C mutant of trastuzumab heavy chain (HC-A114C) was found to be a suitable one for ADC production in terms of conjugation efficiency, low aggregation, and greater stability of the payload in vivo. In later studies, residues near the hinge region [162], in silico-selected solvent-accessible residues [163], and all non-cysteine amino acids of trastuzumab heavy and light chains [164] were individually mutated to cysteine, and the ADC characteristics such as DAR, aggregation, and/or plasma stability were evaluated. From the extensive screening of these mutants, it has been suggested that the reactivity of the engineered thiols and the stability of the linkage are influenced by factors including the conjugation chemistry (e.g., maleimide or disulfide), the position of the introduced cysteines, hydrophobicity of the ADC (which is, in part, correlated with linker exposure to solvent), and the presence of basic amino acids in the vicinity (see above).

Unnatural amino acids can be introduced to recombinant proteins by a number of methods, most notably amber codon suppression by the introduction of an orthogonal pair of tRNA/aminoacyl tRNA synthetase to host cells [165]. Cytotoxic payloads can be conjugated to antibodies site-specifically by the incorporation of unnatural amino acids capable of bio-orthogonal chemical reactions. The unnatural amino acid *p*-acetylphenylalanine, for example, can form an oxime linkage with a linker containing a hydroxylamine group [166]. An ADC with oxime linkage showed a higher serum stability, lower toxicity, and antitumor efficacy in vivo comparable to the maleimide-conjugated ADC [154]. Click reactions, highly specific, one-pot chemical reactions between two bio-orthogonal moieties in aqueous phase [167], are also useful for conjugating linker-payloads to antibodies incorporating unnatural amino acids. *N*^6^-((2-azidoethoxy)carbonyl)-L-lysine, for example, can form a highly stable triazole linkage with alkyne-containing linkers [168]. These reactions are highly efficient with >95% coupling yield, which translates into DAR ~1.9 assuming one unnatural amino acid introduced per half-antibody.

Selenocysteine (Sec), a natural non-canonical amino acid, can be incorporated to recombinant antibodies and conjugated to linker-payloads [169,170]. Selenocysteine remains nucleophilic at weakly acidic pH (p*K*_a_ = 5.2), while cysteine does not (p*K*_a_ = 8.3), making feasible the site-specific conjugation of cytotoxic payloads to antibodies. In selenoproteins, Sec is incorporated near the C-terminus of the protein and encoded by opal (UGA) stop codon. The incorporation of Sec instead of the termination of translation requires a selenocysteine insertion sequence (SECIS) element in the 3′-UTR; however, even with the presence of SECIS, competition between Sec insertion and termination results in a mixture of Sec-incorporated and prematurely terminated polypeptides. This results in predominantly heterodimeric IgG or Fab with selenocysteine inserted in only one of the polypeptide chains (maximum DAR = 1). Despite the limitations of lower production yield than conventional antibodies and low DAR, selenoantibodies offer some interesting possibilities in ADC development. For example, because of the wide difference in p*K*_a_ values between selenocysteine and cysteine, a selenoantibody with additionally engineered cysteine residues can be sequentially conjugated to two different types of linker-payloads [171].

Linker-payload moieties can also be attached to the antibody by enzymatic reactions (Figure 4e). Formylglycine generating enzyme, transglutaminase, sortase, glycosyltransferases, and farnesyltransferase are examples of enzymes used for the conjugation. Formylglycine generating enzyme converts the cysteine in substrate sequences such as CXPXR motif to formylglycine [172]. The resulting aldehyde group is compatible with a number of bio-orthogonal coupling reactions such as coupling with aminooxy or hydrazide functional groups [172], or hydrazine-iso-Pictet-Spengler ligation [173,174]. Transglutaminases couple the glutamine residue in substrate sequences with primary amine; linker-payloads are conjugated to the glutamine side chain through amide bonds [175,176,177]. Sortase catalyzes the ligation of LPXTG tag at the C-terminus of a polypeptide with the N-terminal oligoglycine of another [178]. The C-terminal glycine is removed by the enzyme to form acyl-enzyme intermediate, which then reacts with N-terminal primary amine of the oligoglycine substrate. Glycotransferases can be engineered to transfer modified monosaccharide units to the *N-*glycan of IgG, which are subsequently coupled bio-orthogonally to linker-payloads [179]. Unlike most other site-specific conjugation methods, the glycotransferase method does not require antibody sequence modification, although the *N*-glycan functions may be disrupted by the conjugation. Farnesyltransferase prenylates the cysteine residue in the C-terminal CAAX tag [180,181]. Functionalized prenyl pyrophosphates can be attached to the CAAX tag for ADC production [182]; however, the introduction of hydrophobic prenyl group may affect the solubility of the ADC. While some of these enzymes can catalyze the coupling reaction with high efficiency and specificity, they tend to have a number of drawbacks, including a long substrate sequence attached or inserted to the antibody, a long reaction time, a low yield due to the reversible reaction, a large amount of the enzyme required, and/or byproduct generation [183].

## 4. Concluding Remarks

After the clinical proof of monoclonal antibodies as a valid therapeutic modality in 1980s and 1990s, efforts to improve the efficacy and broaden the mode of action of therapeutic antibodies have led to the successful development of gemtuzumab ozogamicin (approved by FDA in 2000) and catumaxomab (approved by EMA in 2009). These early examples of ADC and bsAb, respectively, were later withdrawn from the market in part due to limited efficacy and/or excessive toxicity (although gemtuzumab ozogamicin was reapproved in 2017; see above). However, advances in the antibody engineering technologies allowed the generation of safer, more efficacious ADCs and bsAbs, many of which are in commercial or late clinical development stages and discussed in this article. In spite of the promises offered by these formats, they also pose unique technical challenges, many of which can be addressed by optimizing the production process and the physicochemical properties. However, some of these challenges are inherent to the core concepts of bsAbs or ADCs. These include balancing affinities of individual arms of bsAbs to maximize their therapeutic window [184], achieving synergism by bispecificity [185,186], and minimizing on-target, off-tumor toxicity of these highly potent molecules [187,188]. Future developments in bsAb and ADC fields are expected to solve many of these issues to provide safer, more efficacious therapies for serious diseases with unmet medical needs.

Finally, an interesting development in the field is the combination of bsAb and ADC technologies, or bispecific antibody–drug conjugates (bsADC). For example, a recent study reported that co-administration of HER2×PRLR bsAb with anti-HER2 ADC drastically enhanced the cytotoxic activity of the ADC, and HER2×PRLR bsADC showed a ~100-fold decrease in EC_50_ against the T47D/HER2 cell line relative to anti-HER2 ADC (0.4 nM vs. 40 nM, respectively) [189], due to the rapid internalization and lysosomal trafficking of PRLR that leads to efficient degradation of the ADC and release of the cytotoxic payload. The amalgamation of technological advancements in bsAb and ADC fields, along with a better understanding of cancer and target biology, is expected to produce more innovative cancer therapeutics that can benefit patients with currently intractable diseases.

## Figures and Tables

**Table 1:** Approved and clinical stage bispecific antibodies and antibody–drug conjugates discussed in this review *.

**Bispecific Antibodies**						
**Tradename**	**INN/Codename**	**Technology**	**Targets**	**Indication**		**Status**
Blincyto	Blinatumomab	BiTE	CD19/CD3	B-cell ALL		Approved
Removab	Catumaxomab	Quadroma	EpCAM/CD3	Malignant ascites		Withdrawn (2017)
Hemlibra	Emicizumab	Common LC	FIXa/FX	Hemophilia A		Approved
	AFM11	TandAb	CD19/CD3	NHL, ALL		Terminated
	Duvortuxizumab	DART	CD19/CD3	B cell malignancies		Terminated
	ABT-165	DVD-Ig	DLL4/VEGF	Solid tumors		Phase 2
	Vanucizumab	CrossMab	Ang-2/VEGF	mCRC		Terminated
	Faricimab	CrossMab	Ang-2/VEGF	AMD		Phase 3
	JNJ63709178	DuoBody	CD123/CD3	AML		Phase 1
	JNJ61186372	DuoBody	EGFR/cMET	NSCLC		Phase 1
**Antibody–Drug Conjugates**						
**Tradename**	**INN**	**Linker-Payload**	**Conjugation**	**Target**	**Indication**	**Status**
Mylotarg	Gemtuzumab ozogamicin	hydrazone-calicheamicin	Lysine	CD33	AML	Approved
Kadcyla	Trastuzumab emtansine	SMCC-DM1	Lysine	HER2	Breast cancer	Approved
Adcetris	Brentuximab vedotin	vc-MMAE	Cysteine	CD30	HL, ALCL	Approved
Besponsa	Inotuzumab ozogamicin	hydrazone-calicheamicin	Lysine	CD22	ALL	Approved
Polivy	Polatuzumab vedotin	vc-MMAE	Cysteine	CD79b	DMBLC	Approved
Padcev	Enfortumab vedotin	vc-MMAE	Cysteine	Nectin-4	mUC	Approved
Enhertu	Trastuzumab deruxtecan	ggfg-MMAE	Cysteine	HER2	Breast cancer	Approved
	Anetumab ravtansine	SPDB-DM4	Cysteine	Mesothelin	Mesothelioma	Phase 2
	Depatuxizumab mafodotin	mc-MMAF	Cysteine	EGFR	Solid tumors	Phase 3
	Mirvetuximab soravtansine	SulfoSPDB-DM4	Cysteine	FOLRα	Ovarian cancer	Phase 3
	Rovalpituzumab Tesirine	va-SG3199	Cysteine	DLL3	Solid tumors	Terminated

* Molecules only briefly mentioned in the main text are not included in this table. INN, international non-proprietary name; BiTE, bispecific T-cell engager; ALL, acute lymphoblastic leukemia; TandAb, tandem diabody; NHL, non-Hodgkin lymphoma; DART, dual affinity retargeting; DVD-Ig, dual variable domain-immunoglobulin; mCRC, metastatic colorectal cancer; AMD, age-related macular degeneration; AML, acute myeloid leukemia; NSCLC, non-small cell lung cancer; SMCC, succinimidyl 4-(*N*-maleimidomethyl)cyclohexane-1-carboxylate; DM, derivative of maytansine; HL, Hodgkin lymphoma; ALCL, anaplastic large cell lymphoma; vc, Valine-Citrulline linker; MMAE/F, monomethyl auristatin E/F; DLBCL, diffuse large B cell lymphoma; mUC, metastatic urothelial cancer; ggfg, Gly-Gly-Phe-Gly linker; SPDB, *N*-succinimidyl-4-(2-pyridyldithio)butanoate; va, Valine-Alanine linker.
